# Reactive-Oxygen-Species-Mediated *P. aeruginosa* Killing Is Functional in Human Cystic Fibrosis Macrophages

**DOI:** 10.1371/journal.pone.0071717

**Published:** 2013-08-19

**Authors:** Noemi Cifani, Barbara Pompili, Marco Anile, Miriam Patella, Daniele Diso, Federico Venuta, Giuseppe Cimino, Serena Quattrucci, Enea Gino Di Domenico, Fiorentina Ascenzioni, Paola Del Porto

**Affiliations:** 1 Department of Biology and Biotechnology “Charles Darwin” Sapienza University, Rome, Italy; 2 Department of Thoracic Surgery, Policlinico Umberto I, Sapienza University, Rome, Italy; 3 Department of Pediatrics and Infant Neuropsychiatry, Centro di Riferimento Fibrosi Cistica Regione Lazio, Sapienza University, Rome, Italy; University of Duisburg-Essen, Germany

## Abstract

*Pseudomonas aeruginosa* is the most common pathogen for chronic lung infection in cystic fibrosis (CF) patients. About 80% of adult CF patients have chronic *P. aeruginosa* infection, which accounts for much of the morbidity and most of the mortality. Both bacterial genetic adaptations and defective innate immune responses contribute to the bacteria persistence. It is well accepted that CF transmembrane conductance regulator (CFTR) dysfunction impairs the airways-epithelium-mediated lung defence; however, other innate immune cells also appear to be affected, such as neutrophils and macrophages, which thus contribute to this infectious pathology in the CF lung. In macrophages, the absence of CFTR has been linked to defective *P. aeruginosa* killing, increased pro-inflammatory cytokine secretion, and reduced reactive oxygen species (ROS) production. To learn more about macrophage dysfunction in CF patients, we investigated the generation of the oxidative burst and its impact on bacterial killing in CF macrophages isolated from peripheral blood or lung parenchyma of CF patients, after *P. aeruginosa* infection. Our data demonstrate that CF macrophages show an oxidative response of similar intensity to that of non-CF macrophages. Intracellular ROS are recognized as one of the earliest microbicidal mechanisms against engulfed pathogens that are activated by macrophages. Accordingly, NADPH inhibition resulted in a significant increase in the intracellular bacteria survival in CF and non-CF macrophages, both as monocyte-derived macrophages and as lung macrophages. These data strongly suggest that the contribution of ROS to *P. aeruginosa* killing is not affected by CFTR mutations.

## Introduction

Cystic fibrosis (CF) is the most common genetic disorder affecting the Caucasian population. This disease is caused by mutations in the CF transmembrane conductance regulator (CFTR), which encodes a cAMP-dependent chloride channel. These mutations lead to malfunction of this chloride channel in CF patients [1], [2]. To date, more than 1,600 mutations in the CFTR gene have been identified [3]. CF is a multi-organ disease that affects the airways, pancreas, small intestine, liver, reproductive tract, and sweat glands [4]. The clinical symptoms are viscid mucus, respiratory infections, intestinal malabsorption of fat, diabetes mellitus, meconium ileus, impaired liver function, male infertility, and salt loss. However, airways infection and inflammation dominate and contribute significantly to the morbidity and mortality associated with CF disease [5]. From early childhood, the CF airways are infected by a restricted range of pathogens, which include *Pseudomonas aeruginosa, Burkholderia cepacia* and *Achromobacter xylosoxidans*. Although repeated and intensive antibiotic therapy contributes to the eradication of *P. aeruginosa*, which is the major bacterial pathogen in CF, this pathogen recurs and eventually develops into a chronic infection [6]. This chronic infection can result in a prolonged inflammatory response, which is believed to cause the tissue injury that leads to progressive loss of lung function [7], [8].

Immune cells assist in the host responses to infections, through pathogen neutralisation and return to homeostasis. It is well accepted that defective CFTR function affects the contribution of the airways epithelium to lung innate immunity [9], [10]. However, a growing body of evidence suggests that other innate immune cells, such as neutrophils and macrophages, are directly affected by CFTR dysfunction, thus contributing to the infectious pathology in the CF lung [11]. In support of this, it was demonstrated recently that conditional inactivation of CFTR in myeloid cells results in a significant increase in bacterial survival and inflammation in mice challenged intra-tracheally with *P. aeruginosa* [12].

In macrophages, the absence of CFTR has been linked to increased pro-inflammatory cytokine secretion and defective *P. aeruginosa* killing [13]–[16]. In the murine model, it has been proposed that impaired bacterial killing results from the failure of CF alveolar macrophages to correctly acidify their degradation compartments, thus compromising pathogen killing [14], [15]. In addition, pH alterations in intracellular vesicles has been proposed to affect the activity of enzymes involved in ceramide metabolism, thus impairing the formation of ceramide-enriched membrane platforms, which in turn affects NADPH oxidase assembly and activity [13]. These explanations are based on the assumption that CFTR serves in the counter-ion pathway involved in lysosomal acidification. However, further studies in CFTR-deficient macrophage cell lines and primary mouse and human alveolar macrophages have failed to reveal pH changes in the phagosomes, which argues against this hypothesis [17], [18].

To date, the role of CFTR activity on macrophage function has been mainly investigated in CF murine models that, contrary to CF patients, are largely spared such over development of lung disease [19], [20]. Only a few studies have examined human macrophages, and no data are available on the activity of the macrophages in other animal models, such pig and ferret. In a previous study, we analysed the bactericidal activity of human macrophages that had been differentiated *in vitro* from monocytes (monocyte-derived macrophages; MDMs). The resulting data demonstrated that macrophages from CF patients have a reduced ability to kill intracellular *P. aeruginosa*, compared to non-CF control macrophages [21].

To obtain further insight into the role of CFTR in macrophage antimicrobial activity in the present study, we extended the analysis of bactericidal activity against *P. aeruginosa* to human lung macrophages, initially confirming an increase in the intracellular bacterial survival in the macrophages carrying dysfunctional CFTR. Then, with the aim to identify the bactericidal mechanism affected by the CFTR mutations, we focused our study on the role of NADPH-oxidase-dependent reactive oxygen species (ROS) in *P. aeruginosa* elimination by human macrophages. Indeed, among the molecular mechanisms and effector molecules relevant to *P. aeruginosa* elimination by macrophages, which include autophagy, asparagine endopeptidase, NO and ROS, this last has been shown to be negatively affected by CFTR mutations in mice [22]–[24]. Thus, we assessed the contribution of ROS to human macrophage activity against *P. aeruginosa*, by evaluating the generation of the oxidative burst and the effects of NADPH oxidase inhibition on the intracellular bacterial survival in non-CF and CF macrophages. Our data demonstrate that NADPH-dependent ROS are involved in the elimination of intracellular *P. aeruginosa* within the first few hours after infection. This activity was detected in both non-CF and CF macrophages, which suggests that this pathway is not affected by CFTR dysfunction. Moreover, we provide evidence that as well as ROS, other non-oxidative mechanisms are involved in the elimination of *P. aeruginosa* by macrophages, as demonstrated by the efficient elimination of these bacteria later in the infection with NADPH-inhibitor treatment of the macrophages.

## Experimental Procedures

### Subjects

Lung macrophages were isolated from 17 patients with CF (9 males, 8 females; median age, 30 years). The genotypes and demographic characteristics of the CF patients are reported in Table 1. The non-CF lung macrophages were isolated from 13 control subjects (6 males, 7 females; median age, 53 years). Samples of lung parenchyma were obtained from patients with CF undergoing double lung transplantation, while lung tissue resections were obtained from non-CF subjects undergoing thoracic surgery. Subjects with chronic obstructive pulmonary disease were excluded from the tissue sampling.

**Table 1:** Characteristics of the CF patients who provided the lung CF macrophages.

Patient	Age	Gender	Genotype
CF13	23	F	F508del/F508del
CF14	30	F	F508del/W1282X
CF15	16	F	F508del/574delA
CF16	34	M	F508del/unknown
CF17	15	M	F508del/F508del
CF18	24	F	F508del/2,3del21Kb
CF19	30	M	F508del/F508del
CF20	35	F	N1303K/H119R
CF21	52	M	F508del/F508del
CF22	30	M	F508del/F508del
CF23	41	M	F508del/F508del
CF24	33	M	F508del/S549R(A_>C)
CF25	39	M	F508del/G542X
CF26	31	F	W1282X/W1282X
CF27	30	M	F508del/N1303K
CF28	28	F	F508del/F508del
CF29	31	F	F508del/G542X

Blood was obtained from 14 healthy donors and from 12 patients with CF, as confirmed by positive sweat tests and detection of CF-inducing mutations (5 males, 7 females; median age, 31 years).

All of the CF patients were clinically stable at the time of blood donation, and were not receiving systemic antibiotic or corticosteroid treatments. Nine of them were F508del homozygous, one was W1282X homozygous, and two carried at least one delta F508del allele (F508del/D192G, F508del/P5L).

All of the patients gave written informed consent, and the study was approved by the local Ethics Committee (*Comitato Etico*, Azienda Policlinico Umberto I, Rome, Italy; 21 June, 2007).

### Isolation of Human Lung Macrophages

Lung macrophages were isolated from lung parenchyma as previously reported [25]. The lung parenchyma was abundantly washed with phosphate buffered saline (PBS) with 0.1% EDTA, and then minced finely with scissors. The dispersed cells were collected and filtered through sterile gauze. These cells were centrifuged at 500×*g* for 10 min, and the cell suspension was enriched in macrophages by flotation in Percoll (GE) density gradients. The recovered macrophage fractions were resuspended in RPMI 1640 (Gibco-BRL, Invitrogren Corporation Carlsbad, CA, USA) supplemented with 5% foetal calf serum, and incubated in 24-well or 48-well multiwell plates (Falcon BD Biosciences) at 37°C in a humidified atmosphere of 5% CO_2_. After an overnight incubation, the adherent cells were detached and stained with an anti-CD68 antibody.

To assess the morphology of the adherent cells, cytospin preparations were made on glass slides by centrifugation at 500× *g* for 2 min. These were stained with a modified Romanowsky stain, using Dif-Stain kits (Titolchimica, Rovigo, Italy) and the images were acquired with a Nikon Eclipse E400 microscope, under the 20× objective.

CFTR expression was analysed on RNA samples isolated from non-CF macrophages using real-time PCR, as previously reported [21].

### Isolation and Differentiation of Human Monocytes

For the preparation of MDMs, peripheral blood mononuclear cells were isolated and differentiated to macrophages, as described previously by Del Porto *et al*. [21].

### Intracellular Staining for CD68

The cells (0.3×10^5^) were washed in PBS and then resuspended in 100 µl cytofix/cytoperm solution (BD, Bioscience) for 20 min at 4°C. The cells were then washed in PBS, and an anti-CD68-FITC antibody (AbD Serotech) in PBS was added for 30 min at 4°C. Cells incubated with the IgG1-FITC isotype in PBS served as controls. The cells were then washed and resuspended in PBS before flow cytometry analysis (FACS Calibur), using the CellQuest software (Becton Dickinson) [26].

### Measurement of Reactive Oxygen Species

Intracellular ROS was detected using 5-(and-6)-chloromethyl-2′,7′-dichlorodihydrofluorescein diacetate, acetyl ester (CM-H_2_DCFDA; Invitrogen). Briefly, the macrophages were incubated with 4 µM CM-H_2_DCFDA in Hank’s balanced salt solution for 30 min at 37°C. After this incubation, the cells were washed and infected in the same buffer with *P. aeruginosa* ATCC 27853 at a multiplicity of infection of 20 (i.e., 20 bacteria per macrophage) for 1 h at 37°C in the absence or presence of the flavoprotein inhibitor diphenylene iododium (DPI, Sigma) at the final concentration of 5 µM. Post-infection, the cells were centrifuged for 5 min at 500× *g*, resuspended in PBS, and stained with propidium iodide before flow cytometer analysis (FACS Calibur), using the CellQuest software (Becton Dickinson).

### Bactericidal Assay

The bactericidal assay was performed as previously reported [27], [28], with the following modifications. Briefly, the day before infection, the macrophages were seeded in 48-well plates in antibiotic-free RPMI medium. The macrophages were then infected with *P. aeruginosa* strain ATCC 27853 (Pa27853) at a multiplicity of infection of 20–30.

The bacteria were brought in contact with the macrophages by centrifugation (500×*g* for 10 min), and the end of this centrifugation was considered as the start of the infection, which proceeded for 1 h at 37°C in a humidified atmosphere of 5% CO_2_. After gentle washing, the extracellular bacteria were killed with 400 µg/ml gentamycin for 1 h. The end of this step was defined as t_0_. The macrophages in selected wells were lysed, to determine the number of intracellular bacteria (CFU) at t_0_; the cells in the remaining wells were incubated in antibiotic-free medium, and selected cell samples were lysed at 2 h (t_2_) and 4 h (t_4_) after t_0_. The fraction of internalised bacteria was determined as the CFU at 0 min after infection (i.e., at t_0_) divided by the input CFU, expressed as a percentage. The bacteria survival was determined as the CFU at 2 h and 4 h after infection, divided by the CFU recovered at 0 min after infection (i.e., at t_0_), expressed as a percentage.

Where indicated, the macrophages were pre-treated for 30 min before infection with 10 µM DPI (NADPH oxidase inhibitor) or 100 µM NG-nitro-L-arginine methyl ester (L-NAME; iNOS inhibitor), infected with Pa27853 for 1 h, and treated with antibiotics for 30 min, to kill the extracellular bacteria. The CFU recovered from the control and treated cells were normalised to the input CFU.

The macrophage viability following *P. aeruginosa* infection was determined in selected samples using acridine orange/ethidium bromide staining for 6 h (see Methods S1 and Fig. S1).

### Statistical Analysis

All of the data were collected as means ±standard deviation, unless otherwise stated. Statistical analysis was performed using the GraphPad Prism 4 software (GraphPad Software Inc.), and the statistical tests are indicated in the Figure legends. Significance was defined as *p*<0.05.

## Results and Discussion

### The Bactericidal Activity of CF Lung Macrophages is Severely Compromised

**Figure 1:**
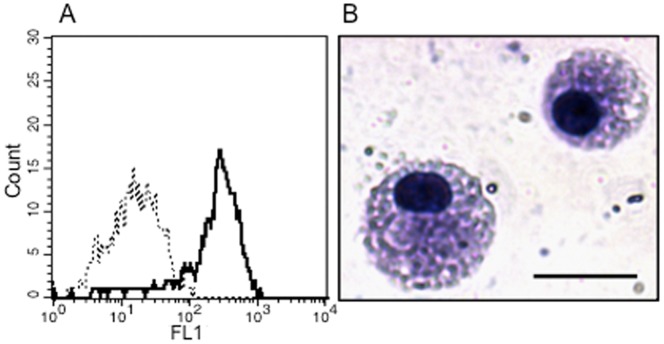
Phenotype of the isolated human lung macrophages. A) Flow cytometric analysis of CD68 expression by lung macrophages stained with anti-CD68-FITC (black line) and a control isotype-FITC (dotted line) antibody. B) Morphology of lung macrophages stained with the Dif-Stain. Scale bar, 20 µm.

To determine whether lung CF macrophages show defective bactericidal activity, similar to *in-vitro* differentiated macrophages, we compared the killing of the intracellular *P. aeruginosa* by the non-CF and CF macrophages, using the antibiotic protection assay over 4 h. The macrophages were isolated from lung parenchyma and identified by morphological features and expression of the macrophage monocytic marker CD68 (Fig. 1). CFTR expression analysis demonstrated similar levels of CFTR transcript in lung macrophages and in MDMs from healthy donors (Fig. S2).

**Figure 2:**
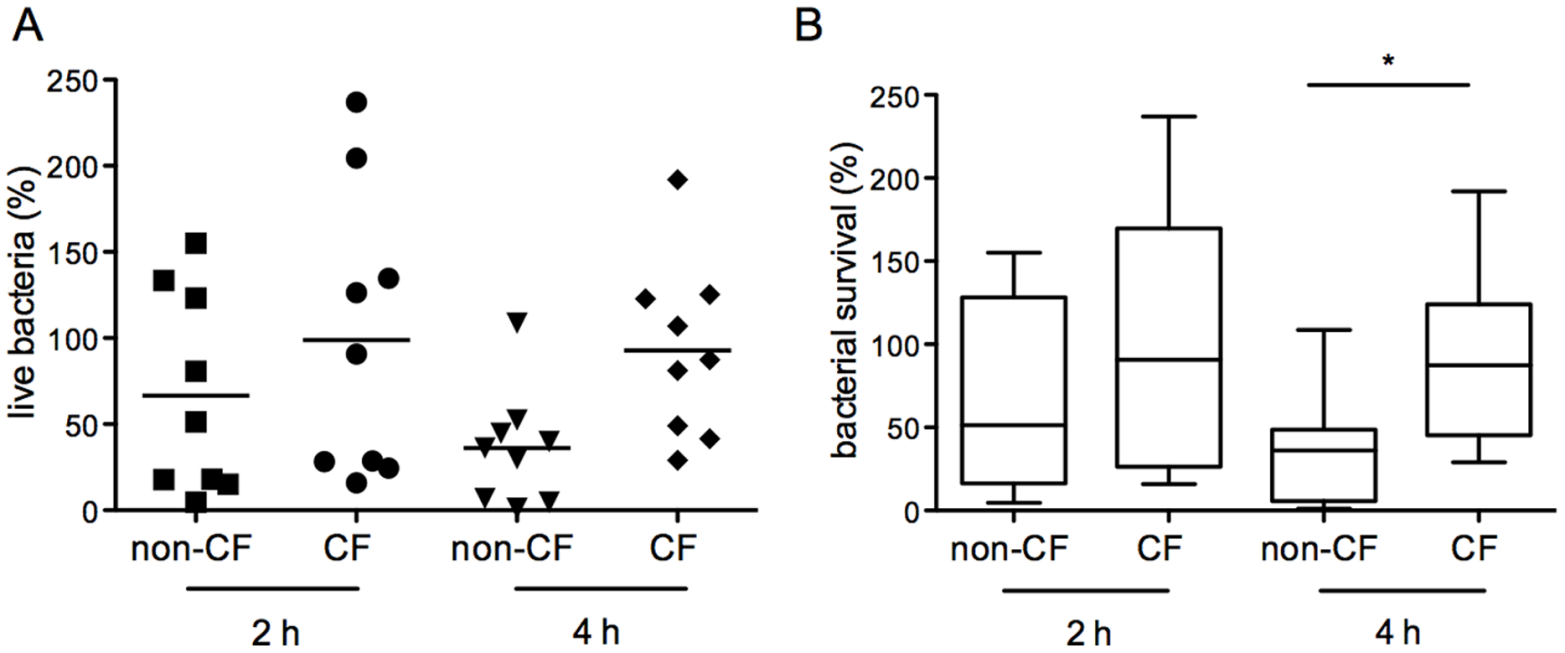
*P. aeruginosa* survival in human lung non-CF and CF macrophages. A) Live intracellular bacteria rescued 2 h and 4 h after infection. Data were normalized to bacteria recovered at the end of the infection (t_0_) set as 100%. Each symbol represents a single subject/patient. The line shows the median percentage live bacteria. B) Box plots of data in A. The 25 and 75 percentiles, median, minimal and maximal are shown. Statistical analysis (Mann Whitney non parametric test), non-CF *vs* CF 2 h and 4 h after infection: *p = *0.297 and *p = 0.0188, respectively.

Macrophages from nine non-CF subjects and nine CF patients were infected with Pa27853 and the live intracellular bacteria were determined 2 h (t_2_) and 4 h (t_4_) after infection. In non-CF macrophages, over this time, the median percentage of surviving bacteria decreased from 66% (t_2_) to 37% (t_4_) (Fig. 2). In contrast, the live bacteria within the lung CF macrophages did not differ significantly over time, with a median survival of 98% and 92% at 2 h and 4 h, respectively, after infection. Statistical evaluation of these data demonstrated that bacteria survival in CF macrophages was significantly higher than in non-CF macrophages at 4 h after infection, and although no significant difference was observed at 2 h, there was a trend to a greater fraction of surviving bacteria in the CF macrophages as compared to the non-CF macrophages. The data variability was relatively high in the CF macrophages, which prompted us to determine the bacteria survival in the macrophages in relation to the CFTR mutations. The F508del homozygous lung macrophages, which accounted for 4 of the 10 samples from the CF patients, showed bacteria survival close to or below the median, whereas for the F508del heterozygous lung macrophages (n = 4), the bacterial survival was above the median (Fig. S3A). However, statistical evaluation of the bacteria survival across the F508del homozygous and F508del heterozygous macrophages failed to reveal significant differences here. We cannot exclude, however, that the analysis of a larger sample size will reveal a correlation between the different CFTR mutations and the macrophage microbicidal activity.

The survival advantage of *P. aeruginosa* in the CF lung macrophages was not explained by differences in phagocytic activity, as the percentage fraction of live intracellular bacteria recovered at the end of the infection (i.e., at t_0_) was similar in the non-CF (58%±0.72%) and CF (47% ±0.84%) macrophages. In agreement with this, it was demonstrated recently that similar percentages of bacteria are phagocytozed by non-CF and CF macrophages isolated from bronchoalveolar lavage samples [29]. Our data thus extend the microbicidal defect previously observed in CF MDMs to lung macrophages.

Overall, the present study and our previous study demonstrate that both blood-derived and lung macrophages isolated from CF patients show significantly reduced killing of intracellular *P. aeruginosa* when compared to the non-CF counterparts. This thus suggests that CF macrophages are intrinsically defective, regardless of the tissue of origin. However, both lung non-CF macrophages and CF macrophages appear less efficient in bacterial killing than MDMs; moreover, the lung CF macrophages showed the greater deficiency. These data partially match data obtained with murine CF models, which showed this microbicidal deficiency in lung but not in peritoneal macrophages. Thus, although CF macrophages appear to be intrinsically defective, the lung environment exacerbates this deficiency.

### Inhibition of NADPH Oxidase Increases Intracellular *P. aeruginosa* Survival in Non-CF and CF Macrophages

**Figure 3:**
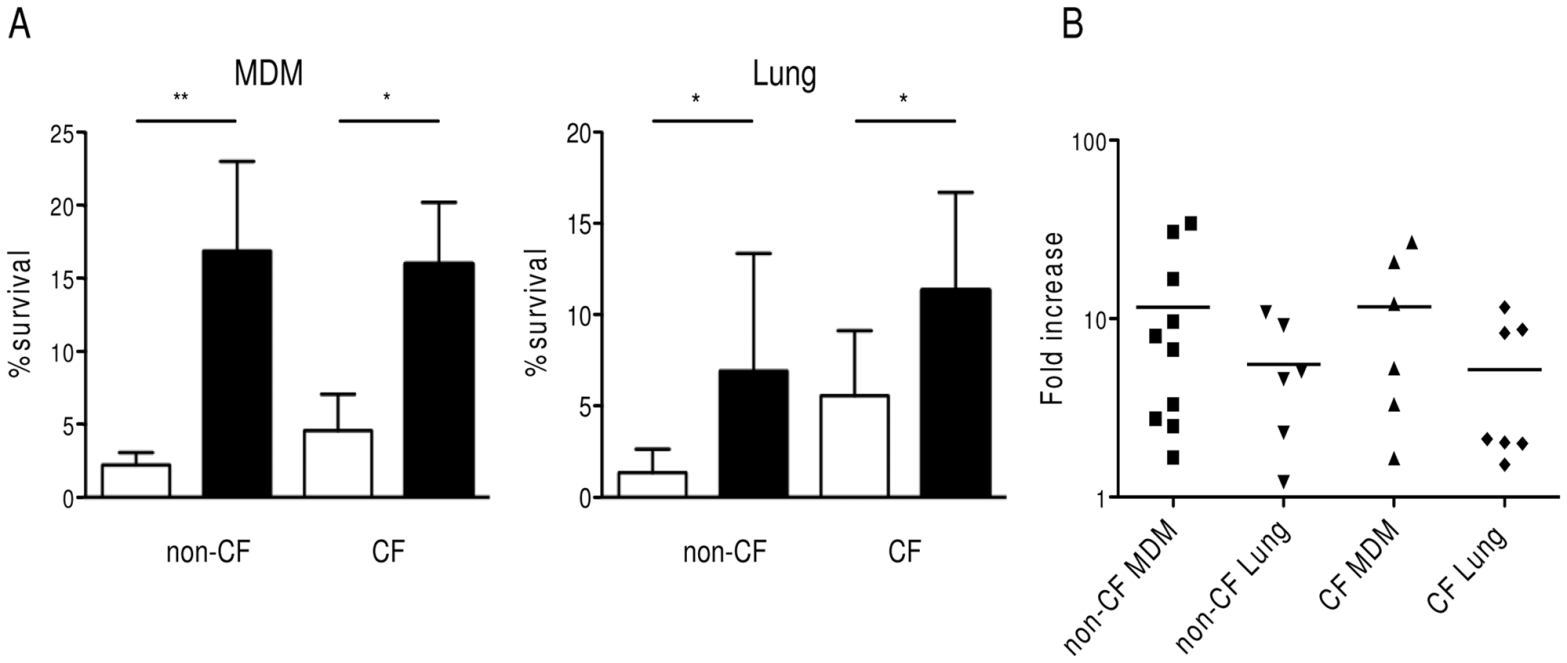
Contribution of ROS to bacterial killing by human non-CF and CF human macrophages. A) Percentage of live intracellular bacteria rescued from untreated (white bar) and DPI-treated (black bar) macrophages. Samples: MDM, monocyte derived macrophages from non-CF subjects (n = 10) and CF patients (n = 6); Lung, macrophages isolated from lung specimens of non-CF subjects (n = 6) and CF (n = 7) patients. Statistical analysis (Wilcoxon non parametric *t*-test) DPI *vs* untreated: non-CF MDMs *p = 0.002; CF MDMs, *p = 0.03; lung non-CF macrophages, *p = 0.031; lung CF macrophages *p = 0.015. B) Fold increase of live intracellular bacteria in DPI-treated macrophages *versus* untreated macrophages. Each symbol represents a single subject, and the line is the mean fold increase.

It has been demonstrated that defective activation of NADPH oxidase leads to impaired ROS production, thus reducing the *P. aeruginosa* killing by *Cftr*-deficient murine alveolar macrophages. Thus, to determine whether this mechanism is also defective in these human macrophages, we analysed the effects of NADPH oxidase inhibition on intracellular *P. aeruginosa* survival in these human non-CF and CF macrophages. For this, MDMs and lung macrophages from non-CF subjects and CF patients were treated with the NADPH oxidase inhibitor DPI prior to the infection with *P. aeruginosa*. The data reported in Figure 3A demonstrate that DPI treatment of these MDMs and lung macrophages leads to significant increases in the survival of the intracellular bacteria in both non-CF macrophages and CF macrophages.

However, the evidence that similar percentages of surviving bacteria were detected in DPI-treated non-CF macrophages and untreated CF macrophages led us to determine whether this might be a consequence of defective ROS generation in the CF macrophages. For this, we quantified the survival advantage of *P. aeruginosa* in NADPH-oxidase-inhibited non-CF macrophages and CF macrophages (Fig. 3B). The ratio of the surviving bacteria in the DPI-treated *versus* untreated macrophages showed a similar increase in bacterial survival in the non-CF macrophages and CF macrophages (mean fold-increase, 6). Similar results were obtained in MDMs, in which the mean increase of bacterial survival was *ca*. 10-fold in non-CF macrophages and CF macrophages. Furthermore, analysis of the fold-increase in bacteria survival in DPI-treated macrophages in relation to CFTR mutations showed a similar distribution regardless of CFTR genotype (Fig. S3B).

These data confirm the importance of ROS-dependent pathways in *P. aeruginosa* killing, and suggest that this pathway is preserved in CF macrophages. However, we cannot exclude that CF macrophages resident in airways can dampen the ROS production in response to the inflammatory milieu.

**Figure 4:**
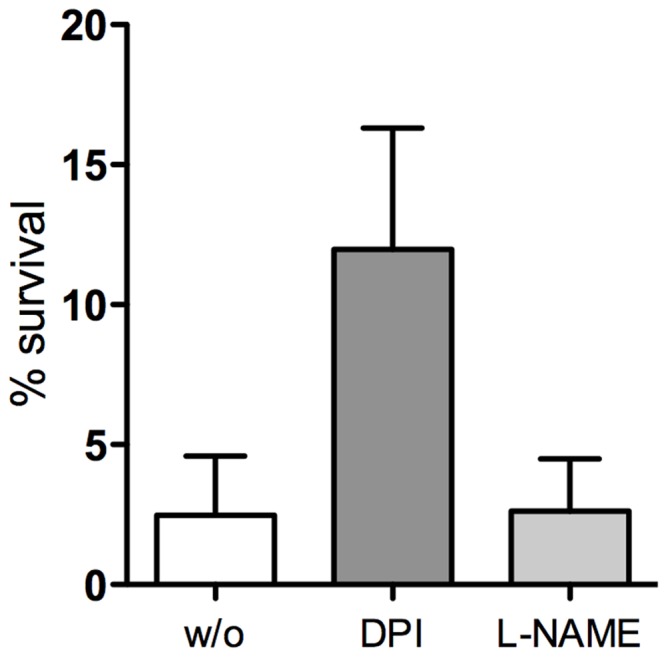
Effect of NADPH and iNOS inhibitors on the survival of *P. aeruginosa* in human macrophages. Intracellular *P. aeruginosa* survival in untreated (w/o) and pre-treated MDMs with either the NADPH oxidase inhibitor DPI or the iNOS inhibitor L-NAME. Data are mean ± SD of three donors, each in triplicate.

As DPI does not distinguish between NADPH oxidase and inducible nitric oxide synthase (iNOS) activity, the inhibition of iNOS was then used to determine whether its activity contributed to bacteria killing at this stage of infection. Pre-treatment of control macrophages with L-NAME, a specific inhibitor of iNOS, had no effect on the bacterial killing by MDMs (Fig. 4).

At variance with the findings with murine CF macrophages [13], our data strongly suggest that CFTR dysfunction does not impair ROS-dependent *P. aeruginosa* killing by human macrophages. This finding is not surprising in view of the major differences in the lung physiology of CF patients and the murine models [20]. Indeed, although the murine models show massive pathological changes in the intestine, they fail to develop the lung disease that is typical of CF patients, possibly due to the low life expectancy and the use of alternative chloride channels, as demonstrated in the lung epithelium [30].

### *P. aeruginosa* Infection Induces an Oxidative Burst in Human Non-CF and CF Macrophages

**Figure 5:**
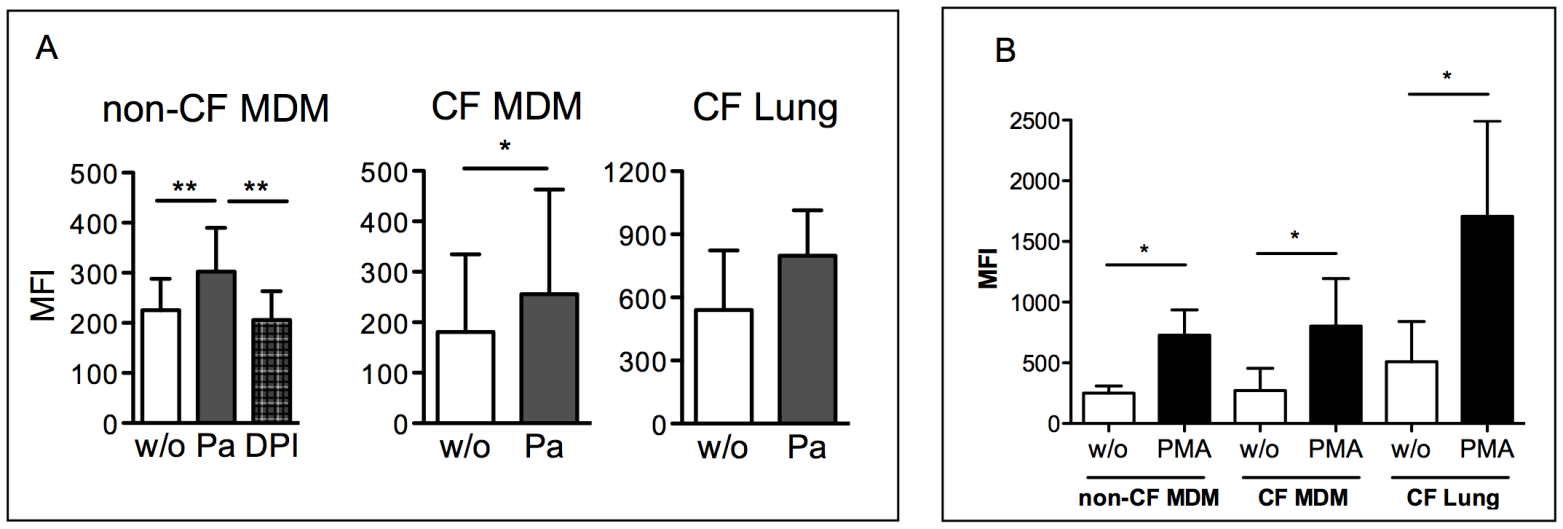
Intracellular ROS production by human control and CF macrophages challenged with *P. aeruginosa*. A) Macrophages loaded with CM-H_2_DCFDA (DCF) were infected with *P. aeruginosa*. Mean Fluorescence Intensity (MFI) of non infected (w/o) or infected (Pa) macrophages was measured by flow cytometry. B) Relative ROS production induced by phorbol 12-myristate 13-acetate (PMA), as detected by the change in fluorescence calculated with the following formula: ΔDCF fluorescence = MFI _infected_^–^ MFI _uninfected_^.^ Samples: monocyte-derived-macrophages (MDM), from non-CF subjects (n = 8) and CF patients (n = 6); lung macrophages from CF patients (CF Lung, n = 3). Statistical significance (Wilcoxon non parametric student’s *t*-test) *p<0.05, **p<0.01.

To further evaluate the functional activity of NADPH oxidase in CF macrophages, we determined the oxidative burst response to *P. aeruginosa* infection. Thus, intracellular ROS production in human non-CF and CF macrophages challenged with *P. aeruginosa* was measured. For this, the macrophages were loaded with the redox sensitive probe CM-H_2_DCFDA (a ROS probe) and then infected with *P. aeruginosa* at a multiplicity of infection of 20 for 1 h. As shown in Figure 5A, *P*. *aeruginosa* infection induced significant increases in fluorescence in the non-CF MDMs, but not in the MDMs treated with the inhibitor DPI, demonstrating that the ROS detected derived from NADPH oxidase activity. Similarly bacterial infection induced ROS production in CF MDMs as well as in lung CF macrophages. In addition, stimulation of macrophages with phorbol 12-myristate 13-acetate, a supra-physiological stimulus, induced similar levels of ROS production in non-CF and CF MDMs. Additionally, lung CF macrophages showed a stronger, although not significant, oxidative burst than for MDMs. Overall, these data further support a lack of any defective NADPH oxidase activity in CF cells (Fig. 5B). This agrees with the conventional paradigm in CF that ROS production by immune cells represents the major cause of airway injury, together with deficient anti-oxidant and anti-protease defences [31], [32].

### NADPH-oxidase-dependent ROS Contribute to Early Killing of *P. aeruginosa* by Human Macrophages

**Figure 6:**
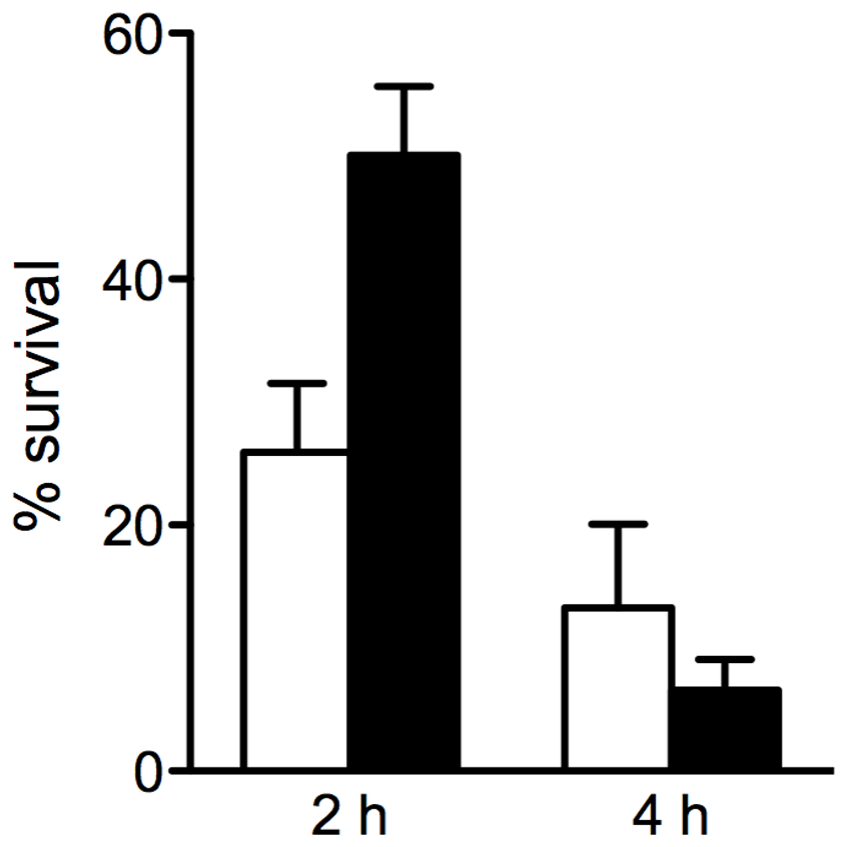
Effects of NADPH oxidase inhibition on intracellular *P. aeruginosa* survival over time. Percentage of live intracellular bacteria rescued from untreated (w/o) or DPI-treated (DPI) human MDMs 2 h and 4 h after infection. Data were normalized to bacteria recovered at the end of infection (t_0_) set as 100%. Data are mean ± SD of three non-CF subjects, each in triplicate.

Having demonstrated that NADPH-oxidase-dependent ROS contribute to the elimination of *P. aeruginosa* in the early phase of macrophage infection, we determined whether this mechanism is responsible for the progressive decrease in the intracellular bacteria viability that is consistently observed in non-CF MDMs as well as in lung macrophages. For this, the survival of the intracellular bacteria was evaluated using antibiotic protection assays in macrophages that were treated or not with DPI over 4 h after infection. The data reported in Figure 6 demonstrate that the bacteria survival advantage in DPI-treated cells persisted for 2 h after infection, with an increase in the percentage of live intracellular bacteria of about twice that in untreated cells. However, no difference in bacteria survival was seen at 4 h after infection, which suggests that as well as ROS production by NADPH oxidase activity, other microbicidal effectors are involved in the elimination of *P. aeruginosa* by human macrophages.

### Conclusions

An increasing body of evidence suggests that CFTR dysfunction affects several components of the innate immunity that predisposes CF infants to infection by the typical bacteria pathogen *P. aeruginosa*. As well as the contribution of epithelial cells to CF lung disease, other immune cells that are either resident or recruited appear to contribute to lung defence. Although they lack the characteristic CF lung disease, murine models, in particular, provide evidence of reduced elimination of intracellular *P. aeruginosa* by macrophages [14], [15] and alterations in macrophage signalling that contribute to elevated inflammatory responses [16]. In line with these observations, we have recently demonstrated that human MDMs from CF patients infected by *P. aeruginosa* show a significant increase in intracellular bacteria survival compared to non-CF cells [21]. Herein, we have further supported this finding by adding a comparative analysis of the intracellular survival of *P. aeruginosa* in human lung non-CF macrophages and CF macrophages, which showed significantly reduced killing of the engulfed bacteria by the CF macrophages.

At present the molecular mechanisms underlying this macrophage deficiency in CF are poorly defined. Murine Cftr-deficient macrophages have been shown to be defective in *P. aeruginosa*-induced ROS production, and inhibition of ROS production in wild-type macrophages mimicked the microbicidal defects of CF macrophages. This suggests that the failure of the CF macrophages to kill *P. aeruginosa* is due to ROS production deficiency [13]. At variance, our data show that either as MDMs or as lung macrophages, human macrophages isolated from CF patients show ROS-dependent microbicidal activity against *P. aeruginosa*. This result is also supported by the finding of a similar oxidative burst response to *P. aeruginosa* infection in non-CF and CF MDMs, which suggests that bacteria-induced ROS production by human macrophages is not influenced by CFTR dysfunction. Similarly, a lack of intrinsic defects in ROS generation was reported for human CF neutrophils isolated from blood samples [33]. However, neutrophils isolated from sputa of CF patients show a reduced respiratory burst to non-bacterial stimulus, possibly due to functional exhaustion [34]. Taken together, these data suggest that human macrophages and neutrophils isolated from CF patients do not have intrinsic dysfunction in induced ROS production, although this activity might be modulated by the lung inflammatory milieu.

Our studies have demonstrated that preservation of ROS-mediated *P. aeruginosa* killing in CF macrophages ultimately does not contribute to bacteria clearance, as suggested by the DPI-treated macrophages, which killed *P. aeruginosa* as efficiently as untreated macrophages 4 h after infection. Interestingly, it has been demonstrated that in human neutrophils, scavenging of superoxide anions and hydrogen peroxide does not alter their killing of intracellular bacteria [35]. In agreement with this, neutrophils isolated from patients with chronic granulomatous disease, which is an inherited defect in NADPH oxidase, effectively kill *P. aeruginosa* [35].

Overall, in the present study, we have demonstrated that the oxidative burst produced by *P. aeruginosa*-infected macrophages contributes to the killing of the intracellular bacteria, and that dysfunction of CFTR does not affect this pathway. Nonetheless, the altered capacity of human CF macrophages to kill *P. aeruginosa* strongly suggests that other pathways are influenced by CFTR mutations. Further studies of the microbicidal mechanisms are required to highlight the role of CFTR in human macrophage functions.

The role of macrophages in the development of lung CF disease is still poorly understood, possibly because the data from CF patients and from animal models have not been uniformly reproduced. However, our studies reported here further support the concept that macrophage dysfunction contributes to lung disease due to their reduced killing of *P. aeruginosa* and their effective production of ROS.

## Supporting Information

Figure S1 **Viability of MDMs from healthy donors following infection with Pa27853 as determined by acridine orange/ethidium bromide staining.**
(PDF)

Figure S2 **CFTR expression by lung macrophages isolated from non-CF patients.**
(PDF)

Figure S3 **Bactericidal activity in relation to CFTR mutations.**
(PDF)

Methods S1 **RNA extraction and RQ-PCR; Acridin orange (AO)/ethidiun bromide (EB) dual staining and fluorescence microscopy.**
(PDF)
