# Supplementary material for: Reactive-Oxygen-Species-Mediated P. aeruginosa Killing Is Functional in Human Cystic Fibrosis Macrophages
Source: PLoS One. 2013 Aug 19;8(8):e71717. doi: 10.1371/journal.pone.0071717 (PMC3747231; doi:10.1371/journal.pone.0071717)
Supplement: Figure S1 — Viability of MDMs from healthy donors following infection with Pa27853 as determined by acridine orange/ethidium bromide staining. (PDF) [file pone.0071717.s001.pdf]

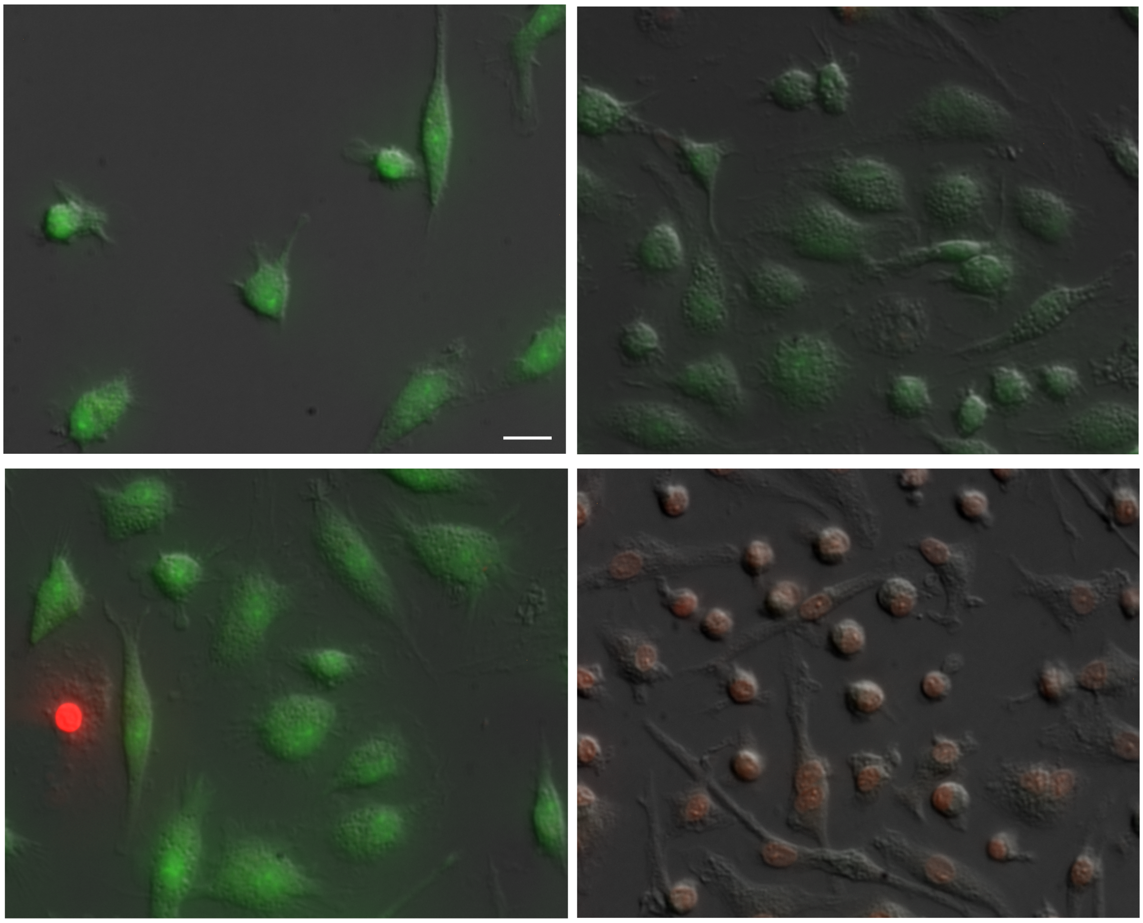

Figure S1. Viability of MDMs from healthy donors following infection with Pa27853 as determined by acridine orange/ethidium bromide staining. Top left, non-infected MDMs; top right, Pa-infected MDMs stained 2 h after infection (t_2); bottom left, Pa-infected MDMs stained 4 h after infection (t_4); bottom right, permeabilized uninfected MDMs (positive control). Scale bar, 25 μm
