# Supplementary material for: Reactive-Oxygen-Species-Mediated P. aeruginosa Killing Is Functional in Human Cystic Fibrosis Macrophages
Source: PLoS One. 2013 Aug 19;8(8):e71717. doi: 10.1371/journal.pone.0071717 (PMC3747231; doi:10.1371/journal.pone.0071717)
Supplement: Figure S2 — CFTR expression by lung macrophages isolated from non-CF patients. (PDF) [file pone.0071717.s002.pdf]

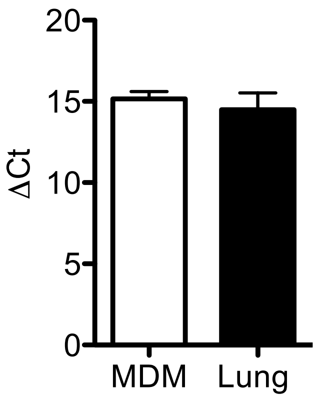

Figure S2. CFTR expression by lung macrophages isolated from non-CF patients. CFTR mRNA expression in macrophages isolated from lung parenchyma (Lung; n = 4) and from MDMs (n = 8), as detected by real-time PCR and quantified by the ΔCt method.
