# Supplementary material for: Reactive-Oxygen-Species-Mediated P. aeruginosa Killing Is Functional in Human Cystic Fibrosis Macrophages
Source: PLoS One. 2013 Aug 19;8(8):e71717. doi: 10.1371/journal.pone.0071717 (PMC3747231; doi:10.1371/journal.pone.0071717)
Supplement: Figure S3 — Bactericidal activity in relation to CFTR mutations. (PDF) [file pone.0071717.s003.pdf]

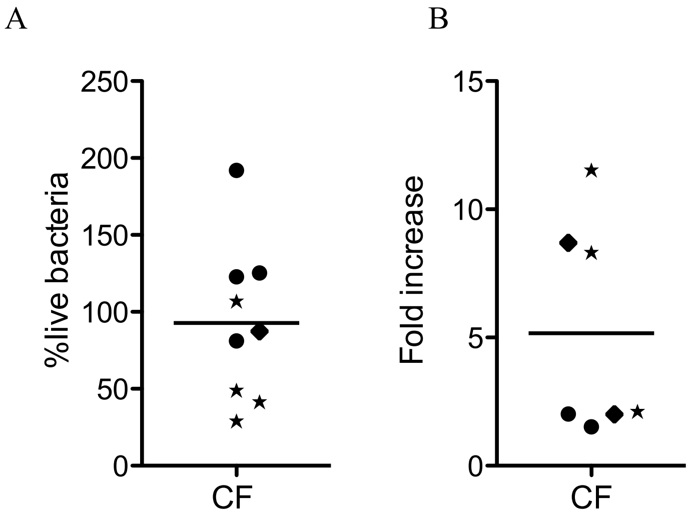

Figure S3. Bactericidal activity in relation to CFTR mutations. A) Live bacteria rescued from lung CF macrophages 4 h after infection. B) Live bacteria in lung CF macrophages treated with DPI, *versus* untreated samples. Stars, F508del homozygous; circles, F598del heterozygous; diamonds, patients lacking F508del mutations (N1303k/H119R in panel A; W1282X/W1282 and N1303k/H119R in panel B).
