# Supplementary material for: Reactive-Oxygen-Species-Mediated P. aeruginosa Killing Is Functional in Human Cystic Fibrosis Macrophages
Source: PLoS One. 2013 Aug 19;8(8):e71717. doi: 10.1371/journal.pone.0071717 (PMC3747231; doi:10.1371/journal.pone.0071717)
Supplement: Methods S1 — RNA extraction and RQ-PCR; Acridin orange (AO)/ethidiun bromide (EB) dual staining and fluorescence microscopy. (PDF) [file pone.0071717.s004.pdf]

Methods S1

RNA extraction and RT-PCR.

Total RNA was extracted as previously reported [1], from small aliquots of lung macrophages ($\leq 10^6$ cells). RNA quality and concentration were determined by electrophoresis and spectrophotometry. Control PCR reactions were performed with standard primers for the endogenous actin gene. Finally, real-time PCR reactions were performed as reported in Del Porto *et al.* [2] and CFTR mRNA quantified by the ΔCt method, with actin used as the endogenous gene.

Acridine orange/ ethidium bromide staining and fluorescence microscopy

Acridine orange/ ethidium bromide dual staining is a rapid and simple method that allows the discrimination between live and dead cells (apoptotic [early and late] and necrotic). Acridine orange stains both live and dead cells, while ethidium bromide is taken up only by cells that have lost their plasma-membrane integrity [3]. Acridine orange/ ethidium bromide stained cells are categorized as live (green) or dead (red).

Briefly, the cells were seeded on 35-mm plates, infected with Pa27853 (multiplicity of infection, 10-20), incubated for 1 h, treated with 400 μg/ml gentamycin for 1 h to kill the extracellular bacteria, washed and incubated in antibiotic-free RPMI medium. The end of antibiotic treatment was marked as t_0. Two hours (t_2) and 4 h (t4) after t_0, the cells were gently washed with PBS, stained with 10 μg/ml acridine orange in PBS, washed with PBS, and then stained with 5 μg/ml ethidium bromide in PBS. Finally the cells were gently washed and rinsed with RPMI medium without phenol red, for microscopy.

Samples were analysed using a Nikon Eclipse Ti inverted microscope equipped with a Plan Fluor 40× (N.A. 0.60, DIC) objective (Nikon) and a DS-Qi1Mc camera (Nikon). Image acquisition was performed using NIS-Elements AR 3.2 (Nikon); images were processed with Adobe Photoshop CS 8.0 and NIS-Elements AR 4.0.

Supporting References

1. Auriche C, Carpani D, Conese M, Caci E, Zegarra-Moran O, Donini P and Ascenzioni F (2002) Functional human CFTR produced by a stable minichromosome. EMBO Rep. **3**: 862-868.

2. Del Porto P, Cifani N, Guarnieri S, Di Domenico EG, Mariggio MA, Spadaro F et al. (2011) Dysfunctional CFTR alters the bactericidal activity of human macrophages against *Pseudomonas aeruginosa*. PLoS One. 6: e19970.

3. Galluzzi L, Aaronson SA, Abrams J, Alnemri ES, Andrews DW et al. (2009) Guidelines for the use and interpretation of assays for monitoring cell death in higher eukaryotes. Cell Death and Differentiation 16: 1093-1107.
